# Molecular Characterization of *NF1* and Neurofibromatosis Type 1 Genotype-Phenotype Correlations in a Chinese Population

**DOI:** 10.1038/srep11291

**Published:** 2015-06-09

**Authors:** Jia Zhang, Hanxing Tong, Xi’an Fu, Yong Zhang, Jiangbo Liu, Ruhong Cheng, Jianying Liang, Jie Peng, Zhonghui Sun, Hong Liu, Furen Zhang, Weiqi Lu, Ming Li, Zhirong Yao

**Affiliations:** 1 Department of Dermatology, Xinhua Hospital, Shanghai Jiaotong University School of Medicine, Shanghai, 200092, China; 2 Department of General Surgery, Zhongshan Hospital, Fudan University, Shanghai, 200032, China; 3 Shandong Provincial Institute of Dermatology and Venereology, Shandong, 250022, China; 4 Department of Dermatology, Bao’an Maternal and Child Health Hospital, Shenzhen, Guangdong, 518000, China; 5 Department of ophthalmology, Xinhua Hospital, Shanghai Jiaotong University School of Medicine, Shanghai, 200092, China; 6 Department of Dermatology, Fengxian Institute of Dermatosis Prevention, Shanghai, 201408, China

## Abstract

Neurofibromatosis type 1 (*NF1*) is an autosomal dominant hereditary disease that is primarily characterized by multiple café au-lait spots (CALs) and skin neurofibromas, which are attributed to defects in the tumor suppressor *NF1*. Because of the age-dependent presentation of NF1, it is often difficult to make an early clinical diagnosis. Moreover, identifying genetic alterations in NF1 patients represents a complex challenge. Currently, there are no effective detective methods, and no comprehensive *NF1* mutation data are available for mainland China. We screened 109 Chinese patients from 100 families with NF1-like phenotypes (e.g., CALs, neurofibromas, etc.) using Sanger sequencing, multiplex ligation-dependent probe amplification and cDNA sequencing. *NF1* mutations were identified in 97 individuals, among which 34 intragenic mutations have not previously been reported. Our exhaustive mutational analysis detected mutations in 89% (89/100) of the NF1-like probands and 93% (70/75) of subjects fulfilling the National Institutes of Health (NIH) criteria. Our findings indicate that individuals who exclusively present with multiple CALs exhibit a high possibility (76%) of having NF1 and show a significantly lower mutation rate (p = 0.042) compared with subjects who fulfill the NIH criteria, providing clinicians with the information that subjects only with multiple CALs harbor a considerable possibility (24%) of being attributed to other comparable diseases.

Neurofibromatosis type 1 (NF1; OMIM 162200) is one of the most common and complex autosomal dominant disorders, with a worldwide prevalence of at least 1 in 4,000^1^. It is clinically characterized by features such as cutaneous neurofibromas, CALs and Lisch nodules, which are present in the majority of patients at puberty^2^.

The National Institutes of Health (NIH) criteria for NF1^3^ are generally accepted for making a diagnosis according to clinical information of a patient. These clinical criteria are highly specific and sensitive in adults with NF1. However, only 50% of the pediatric NF1 patients without a family history meet the criteria for diagnosis by the age of 1 year, whereas almost all do so by the age of 8 years^2^, highlighting the importance of molecular diagnosis.

The *NF1* gene spans approximately 350 kb of genomic DNA and contains 57 constitutive exons and three alternatively spliced exons^4^. The most common transcript encodes a 2818-amino-acid polypeptide, neurofibromin. One vital functional region, a highly conserved GAP-related domain (GRD) encoded by exons 20–27a^4^,^5^, has been identified and well defined.

Mutational analysis of the *NF1* gene has been found to be a major challenge because the gene is extremely large, in combination with the presence of pseudogenes, a lack of mutation hotspots and a wide mutation spectrum, ranging from single nucleotide substitutions to large deletions^6^. To date, 2030 different *NF1* mutations have been reported and listed in the Human Gene Mutation Database (HGMD). The majority of these mutations lead to truncated forms of neurofibromin, and approximately 25–50% of the mutations are expected to result in RNA splicing abnormalities^7^,^8^.

NF1 is characterized by highly variable expression and marked inter- and intrafamilial variation^9^. Few clear correlations have been established other than a whole-*NF1* deletion associated with severe clinical phenotypes, such as cognitive defects, body or facial dysmorphisms, an early onset of cutaneous neurofibromas^10^,^11^, and a 3-bp in-frame deletion (c.2970_2972delAAT) linked to a relatively milder phenotype (with an absence of neurofibromas)^12^.

Numerous studies aimed at the clinical and molecular characterization of *NF1* have been performed in many populations worldwide. However, few of these studies have involved a Chinese background. Lee *et al.* conducted the largest study in 107 NF1 patients of Chinese origin, identifying 45 novel and 23 known *NF1* mutations^13^.

The present study represents the first large genetic study of NF1 patients in mainland China. The aim of the present study is to characterize a wide spectrum of *NF1* mutations and elucidate genotype–phenotype correlations through integrated mutational screening and clinical data collection. The obtained data should consequently provide a simple and effective strategy for early diagnosis and genetic counseling.

## Results

### General description of clinical characteristics and genetic results

The clinical descriptions and mutation data are summarized in Table S1–S4. In total, 89 out of the 100 indexed families harbored pathogenic mutations, consisting of 21 nonsense mutations, 26 frameshift mutations, 15 splicing mutations, 12 missense mutations, 2 in-frame deletions, 1 indel, 11 microdeletions and 1 single-exon deletion. Thirty-four intragenic mutations were novel (see Table S1 and Table S4). The mutations were evenly distributed along the *NF1* coding sequence at first glance. However, after being weighted by exon size, exon 15 was found to show a remarkably high mutation frequency (Figure S1).

There were significantly different mutation rates between subjects who fulfilled the NIH criteria (70/75) and those who only exhibited CALs (19/25) (Chi-square test, p = 0.042).

Mutation analysis of *SPRED1* was performed in 14 index patients without any pathogenic mutations in *NF1*. However, the results were negative.

### Large deletions detected through multiplex ligation-dependent probe amplification (MLPA) analysis

Twenty-seven samples were analyzed via MLPA. Eleven cases with gross *NF1* gene deletions were identified using the MLPA P122 kit and confirmed with the MLPA P081/082 kit. Among these deletions, six were suspected to be the most common type-1 *NF1* deletions, whereas the remaining five were atypical deletions, ranging from 0.6 to 1.7 mb (see Table S4). The MLPA P081 kit revealed an intragenic deletion of exon 28 in an *NF1* family with neurofibromas, which was also confirmed at the mRNA level (data not shown).

It has been reported that breakpoints at PRS1 and PRS2 accounted for 69% of *NF1* type-1 deletions^14^,^15^. Notably, in our cohort, for all six of the patients in whom a type-1 deletion was indicated, a product was generated in PRS2 deletion breakpoint-spanning PCR assays (see Table S4).

### Pathogenic mutations and polymorphisms from genomic DNA (gDNA) and cDNA sequencing

Missense mutations are generally considered to be benign and less likely to affect the function. Among the five novel missense mutations identified (See Table S1), c.737C >A and c.1207C >T were predicted to be benign using Polyphen2, whereas the other three were all indicated to damaging and deleterious. Two mutations (c.1207C >T, c.3211G >C) segregated in the unaffected parents. Unfortunately, the corresponding parental blood samples for the other three probands (c.737C >A, c.746T >C and c.7126G >A) were unavailable. Nonetheless, these mutations were all absent in 100 normal controls, and sequence comparison of *NF1* across different species showed that all of the corresponding amino acid residues are conserved, implying that these mutations are likely to be pathogenic.

Overall, splicing errors were detected in 18/100 (18%) families. Nine of these were these errors occurred at the canonical GT splice donor or AG splice acceptor, while the others pertained to the group of atypical splicing mutations that create novel 5’ or 3’ splice sites, and their pathogenic effects were demonstrated at the mRNA level if available (Table S1).

Some of the observed sequence variations were inconsistent with all of the criteria for pathogenic mutations (Table S5), indicating a high likelihood of polymorphisms.

## Discussion

In the present study, we performed an integrated genetic analysis (sequential Sanger sequencing, MLPA and cDNA sequencing) in a cohort of 109 patients who were clinically suspected to have NF1. RNA-based technique has been demonstrated to effectively detect ‘deep’ intronic mutations, but it did not work in all the six negative patients with available fresh blood in our cohort (after Sanger sequencing and MLPA). Due to the relatively strict recruitment criteria, the overall detection rate (89%) in this study was higher than in previous similar studies employing Sanger sequencing complemented with MLPA^16^,^17^ and in the study with same Chinese background^13^.

Additionally, there were 25 patients with unfulfilled NF1 consensus diagnostic criteria that were included in our study. When this group was excluded, the detection rate rose to 93%, which is the highest mutation detection rate ever recorded for the *NF1* gene using Sanger sequencing and the MLPA technique, meanwhile, comparable to those obtained in combination with cDNA sequencing^18^,^19^. We also tested blood samples from 20 patients (data not shown) with segmental CALs or only two or three CALs as a control during preliminary work. However, no mutations were found, thus verifying the importance of the recruitment criteria. Notably, the effectiveness of the NIH diagnostic criteria for NF1 was greatly decreased for patients under 4 years of age (Table S2). We also identified 19 mutations in 25 patients who did not meet the NIH criteria, highlighting the importance of molecular diagnosis. The distinct mutation rates between the subjects who fulfilled the NIH criteria and those only with CALs suggest that the latter group may be have other comparable diseases (approximately 24%), rather than NF1.

Many studies involving microdeletions in NF1 patients have estimated the occurrence to be approximately 5–10%^20^, with the most common type (type-1 deletions) accounting for 60–70%. The proportion of microdeletions in this cohort was 11%. Among these deletions, six were type-1 deletions with breakpoints at PRS2, suggesting that the MLPA technique used in this study is reliable and very likely detected all large deletions. Five atypical deletions should be investigated further using flanking SNPs and microsatellite markers, or a high-resolution technique[such as array comparative genomic hybridization (CGH)^21^] to accurately determine how many genes were present within the deletion intervals.

We observed that frameshift mutations accounted for the largest proportion of the identified mutations (30%). The majority of these mutations were *de novo* and novel, particularly for 1-bp deletions, which is in agreement with the LOVD database and previous reviews^19^,^22^. These results imply that small deletions and insertions is prone to occur spontaneously as a consequence of the high mutation rate and potential modifying factors of *NF1.*

Messiaen *et al.* reported that approximately 30% of pathogenic *NF1* mutations influence mRNA splicing and that approximately 30%–50% of all such mutations are located in the non-consensus splice-regulatory sequences^8^,^19^. The cDNA sequencing approach combined with review of the LOVD database and previous reports showed that 20/89 (22.4%) of the identified mutations disturbed *NF1* transcript splicing in our cohort. This relatively low splice mutations ratio^18^,^19^ may have occurred because we were unable to perform analysis at the RNA level in several unidentified patients without fresh blood samples. Previously reported bioinformatics assessments^8^,^19^ could be further applied to reveal how authentic splice sites and hidden regulatory sequence motifs (e.g., exonic splicing enhancers/silencers) are interfered with these exonic and intronic variants at non-consensus splice sites.

The method applied in this study failed to detect any classic *NF1* mutations in six index patients with clear NF1 phenotypes that fulfilled the NIH diagnostic criteria as well as six subjects with suspected NF1 phenotypes. Considering previous results indicating a considerable detection rate of *SPRED1* mutations in *NF1* mutation-negative families with autosomal dominant inheritance of multiple CALs (with or without freckling) and no other features of NF1^23^, Sanger sequencing and MLPA for *SPRED1* were performed. However, no pathogenic mutations were found. With the exception of the existence of deep intronic mutations in *SPRED1* and *NF1*, some of these mutation-negative patients most likely harbor a segmental or mosaic variant of the *NF1* and the affected tissue must be therefore analyzed to identify the mutation. Others may be disorders that overlap with NF1, such as LEOPARD syndrome or neurofibromatosis-Noonan syndrome^24^,^25^. Unfortunately, corresponding tissues and tests were not available in this study. These remaining negative cases require long-term follow-up and more intensive investigations, including RNA test in short-term cultured lymphocytes or the application of next-generation sequencing approach to search for causal mutations in other loci of *NF1* or the entire human genome.

High-signal lesions on T2-weighted brain MRI, termed unidentified bright objects (UBOs), were observed in 55% of the patients. They were particularly common in those aged between 3 and 11 years and were infrequent in children less than 3 years of age, similar to a previous report^26^. These unidentified lesions are proposed to be due to spongiform myelinopathy or vacuolar changes in myelin filled with water^27^, which may be transient or intermittent^28^,^29^. UBOs occur frequently in patients with NF1, but in our cohort, adding it as a new diagnostic criterion only permitted a clinical diagnosis in one of four suspected patients. We also observed UBOs in three mutation-negative patients with only segmental CALs, suggesting that the diagnostic value of UBOs in pediatric patients who only exhibit CALs is worthy of discussion.

Lisch nodules were present in 65.4% of the patients. The majorities were bilateral, and the numbers appeared to exhibit an age-dependent tendency (data not shown), as previously reported^30^. The median ages of NF1 patients with or without Lisch nodules were 6 and 4 years, respectively. Moreover, nearly all of the patients with Lisch nodules harbored *NF1* mutations. Therefore, similar to neurofibromas, Lisch nodules are an extremely effective diagnosis criterion for NF1, particularly in patients over 6 years old.

Malignancies were observed in eight mutation-positive cases (See Table S3). Notably, there were no whole-*NF1* deletions detected.

In our study, the patients with anemic nevus (n = 4), juvenile xanthogranulomas (n = 1) or hemangiomas (n = 1) in our study, all had a confirmed molecular diagnosis of NF1, suggesting that these cutaneous signs bear potential diagnostic value, as previously reviewed^31^,^32^.

The index patients with whole-*NF1* deletions were found to exhibit a higher incidence of neuropsychological abnormalities (2/4) and osseous lesions (5/11), but not an obvious early onset of cutaneous neurofibromas (median age: 6 versus 7 years), roughly in accordance with the previous studies^10^,^11^. We did not observe clearly milder NF1 phenotypes in patients with missense mutations (the four mutations inducing splice error were excluded) in our cohort, which can be explained by the fact that some of these mutations may lead to splicing errors that truncate the tumor suppressor protein and result in relatively severe phenotypes.

The GRD region has been proposed to be hot spot for missense mutations^5^,^33^. However, in the present study, intragenic mutations or missense mutations were not observed to be significantly increased in this functional domain. Nevertheless, exon 15 is prone to be a mutation hotspot and shows potential for further research in Chinese populations (Figure S1).

In the present study, we performed molecular analyses of the *NF1* gene in 109 patients with variable clinical phenotypes. Compared with the findings of Lee *et al.*, the proportion of mutation types and clinical features were similar, with the exception of Lisch nodules (65.4% versus 4.4%). Mutations c.574C >T, c.1318C >T, c.3826C >T and c.7486C >T are shared in both studies, but they are also common in other studies (Table S1). Our study adds to the notion that there are no obvious mutation hotspots in *NF1*, and we were unable to find any novel clear relationships between the type and locus of mutations and distinct clinical features. Phenotypic differences in NF1 patients are more likely to be caused by mechanisms such as “a second hit”, modifying genes that are unlinked to the *NF1* locus, epigenetic alterations or other environmental factors^9^,^34^,^35^. Due to a lack of some detailed clinical information, our investigation regarding the correlations between genotypes and phenotypes was limited. However, our exhaustive mutational screening method can still serve as a cost-effective diagnostic strategy with high sensitivity in the setting of Chinese patients suspected of NF1, but without adequate clinical features corresponding to the NIH diagnostic criteria.

Neurofibromas and other complications result in a strong physical and mental burden for NF1 patients. There are currently still no effective drug treatments for neurofibromas, which highlights the necessity of prenatal diagnosis. Further prenatal analyses of these patients will be available based on their molecular data.

This is the first relatively large NF1 study conducted on the Chinese mainland, and by evaluating the detection rate, we preliminarily confirmed that the methods we used were effective for revealing pathogenic mutations in patients suspected of NF1 (especially for those pediatric patients who only exhibit with CALs). Further research should be conducted to study this common inherited disease, particularly for patients who are negative in comprehensive genetic analyses of *NF1*. Thus, we will be able to establish optimized strategies for early diagnosis and provide genetic counseling in the Chinese population.

## Methods

This study was approved by the Institutional Review Board of Xinhua Hospital, Shanghai JiaoTong University School of Medicine and was conducted in accordance with the principles of the Declaration of Helsinki. All participants gave their written informed consent.

The enrolled patients were tested in order via Sanger sequencing, MLPA and cDNA sequencing (See Appendix S1). Sequence variants were classified as pathogenic mutations by following one of the criteria listed in the supplemental data.

## Additional Information

**How to cite this article**: Zhang, J. *et al.* Molecular Characterization of *NF1* and Neurofibromatosis Type 1 Genotype-Phenotype Correlations in a Chinese Population. *Sci. Rep.* **5**, 11291; doi: 10.1038/srep11291 (2015).

## Supplementary Material

Supplementary Information
