# Supplementary material for: Molecular Characterization of NF1 and Neurofibromatosis Type 1 Genotype-Phenotype Correlations in a Chinese Population
Source: Sci Rep. 2015 Jun 9;5:11291. doi: 10.1038/srep11291 (PMC4460887; doi:10.1038/srep11291)
Supplement: Supplementary Information [file srep11291-s1.pdf]

Molecular Characterization of *NF1* and Neurofibromatosis Type 1 Genotype-Phenotype Correlations in a Chinese Population

Jia Zhang[1], Hanxing Tong[2], Xi'an Fu[3], Yong Zhang[2], Jiangbo Liu[4], Ruhong Cheng[1], Jianying Liang[1], Jie Peng[5], Zhonghui Sun[6], Hong Liu[3], Furen Zhang[3], Weiqi Lu[2#] ,Ming Li[1#], Zhirong Yao[1#]

1.Department of Dermatology, Xinhua Hospital, Shanghai Jiaotong University School of Medicine, Shanghai, 200092，China

2.Department of General Surgery, Zhongshan Hospital, Fudan University, Shanghai, 200032, China

3.Shandong Provincial Institute of Dermatology and Venereology, Shandong, 250022, China

4.Department of Dermatology, Bao'an Maternal and Child Health Hospital, Shenzhen, Guangdong, 518000,China

5.Department of ophthalmology, Xinhua Hospital, Shanghai Jiaotong University School of Medicine, Shanghai, 200092,China

6.Department of Dermatology, Fengxian Institute of Dermatosis Prevention, Shanghai, 201408, China

Jia Zhang and Hanxing Tong contribute equally to this work.

#Correspondence: Zhirong Yao, MD, Department of Dermatology, Xinhua Hospital, Shanghai Jiaotong University, School of Medicine, 1665 Kongjiang Road, Shanghai 200092,China. Tel.:86-21-25078570 Fax: 86-21-65030840, E-mail: zryaosmu@sohu.com

Ming Li, MD, Department of Dermatology, Xinhua Hospital, Shanghai Jiaotong University, School of Medicine, 1665 Kongjiang Road, Shanghai 200092, China. E-mail: aypyslm@163.com

Weiqi Lu, MD, Department of General Surgery Zhongshan Hospital, Fudan University 180 Fenglin Road, Shanghai, 200032, China. Tel: 86-21-64041990 Fax: 86-21-64041990 E-mail: lu.weiqi@zs-hospital.sh.cn

Supplementary information

Supplementary materials and methods:

Patients

The clinically definite or suspected NF1 patients came from 5 hospitals. The suspected NF1 patients who were ultimately enrolled for the study had to strictly meet one of the NIH diagnostic criteria: showing more than 6 CALs (>0.5 cm in childhood, >1.5 cm in puberty), as verified by experienced dermatologists. (Because many clinical features among the NIH diagnostic criteria develop with age, with the exception of CALs, which can commonly present in infancy, so children without a family history generally do not fulfill the NIH diagnostic criteria before 1 year of age.) A total of 109 patients from 100 families were recruited in this cohort from January, 2010 to July, 2014. The median age was 13 years (ranging from 2 months to 60 years). The F/M Sex ratio was close to 1 (see Supplement Table S5). Among the study subjects, eighty-four individuals definitely fulfilled the NIH diagnostic criteria, and twelve probands in this cohort had a family history, and their affected family members (except NF026, NF032 and NF033) were also recruited.

DNA and RNA extraction

There were two dominant types of peripheral-blood samples evaluated in our study. Fresh samples, mainly from the outpatients at Xinhua Hospital (the unaffected parents of the subjects were also recruited if it is available.), and the 'aged' ones from the other four hospitals. Genomic DNA (gDNA) was extracted for Sanger sequencing and MLPA.

Each fresh peripheral-blood samples was divided into two parts: one to be used for DNA extraction (Sanger sequencing and MLPA) and the other for RNA extraction (RT-PCR and cDNA sequencing).

Sanger sequencing

As a first step, we designed primers flanking all coding exons and intron-exon boundaries of the *NF1* gene using Primer Premier 5.0 software (see Table S6). The gDNA samples were subsequently amplified through polymerase chain reaction (PCR), and PCR products were purified and directly sequenced using an ABI PRISM®3730 automated sequencer (Applied Biosystems, USA). In addition, samples from available unaffected parents and 100 unrelated population-match controls were sequenced for the detected mutation to exclude the possibility of polymorphisms in the *NF1* gene.

MLPA analysis

When no pathogenic mutations were found via Sanger sequencing, the patients' gDNA samples were analyzed using SALSA MLPA kits P122, P081 and P082 (MRC-Holland, Amsterdam, The Netherlands). The MLPA kit (P122 version C1) was used to detect deletions/duplications spanning the entire *NF1* gene and to preliminary evaluate the type of deletions. There are two major types of microdeletions. One consists of 1.4 Mb deletions (type-1 *NF1* deletions), encompassing 14 genes, with breakpoints in the *NF1* low copy repeats, and the other is 1.2 Mb deletions (type-2), which encompassed 13 genes and were mediated by recombination between the *JJAZ1* gene and its pseudogene [1, 2].

Type-1 deletions show a particular characteristic in that the breakpoints cluster within two preferred regions, termed paralogous recombination sites 1 and 2 (PRS1 and PRS2) [3]. Due to the poor positioning of the P122 probes, we used the primers as previously described to detect the breakpoints and determine the number of deleted genes precisely [3].

Peak areas for each separated fragment were measured by using Coffalyser. Net software (MRC-Holland, Amsterdam, The Netherlands
). Ratios of <0.65 and >1.35 were considered to indicate deletions and duplications, respectively. DNA copy-number variations identified through MLPA were confirmed via real-time PCR as previously described [4].

RT-PCR test

Total cellular RNA was directly extracted from available fresh blood samples with RNAiso Plus (TAKARA, Dalian, CHN) according to the manufacturer's instructions.

To verify the possible transcript effect of the detected mutations (especially splice site mutations) detected through Sanger sequencing, RT-PCR tests using specific primers were carried out in part of patients (mostly children without neurofibromas).For the subjects showing no candidate mutations in the Sanger sequencing and MLPA analyses, the entire *NF1* cDNA was divided into 23 overlapping fragments and was amplified via PCR with primers ever published [5].

Sanger sequencing and MLPA for Legius syndrome

The Legius syndrome (OMIM 611431), caused by mutations in *SPRED1* must also be considered in the differential diagnosis of suspected NF1 cases without neurofibromas. Sanger sequencing and MLPA P295 Kit (MRC-Holland, Amsterdam, The Netherlands) for *SPRED1* were performed in 14 index patients who harbored no pathogenic mutations in *NF1* (after mutational analyses through all of the above means).

Description of Mutations

The identified mutations were compared with the corresponding NCBI reference NM_000267.3. The exon number was given according to the conventional rule. Mutations were considered as novel if they had not been reported in the literature, Human Gene Mutation Database (HGMD) and Leiden Open Variation Database (LOVD), which are accessible at (http://www.hgmd.cf.ac.uk/ac/index.php) and (http://www.lovd.nl/NF1), respectively.

Pathogenic mutations: a) Forming a stop codon that truncated the protein (frame shift, nonsense). b) Resulting in a splicing error (consensus or non-consensus splice site). c) They were *de novo* in the patient (not present in the unaffected biological parents and 100 unrelated, ethnically matched healthy controls) and were not referred to as a polymorphism of the human genome in the online database, and the predictions of bioinformatics were prone to be deleterious. d) They were previously reported as pathogenic. Table 1 provides a summary of the identified pathogenic mutation.

Table S1. Functional consequence predictions and mRNA effect of pathogenic mutation identified in this cohort

Location	Nucleotide change	Amino acid change	Variant type	References	mRNA effect	Polyphen2	Sift	Mutation Taster
E08	c.737C>A	p.Ala246Glu	Missense	This report	NA	Benigh	Deleterious	Disease causing
E08	c.746T>C	p.Leu249Pro	Missense	This report	NA	Possibly damaging	Deleterious	Disease causing
E11	c.1207C>T	p.Pro403Ser	Missense	This report	NA	Benigh	Deleterious	Disease causing
E13	c.1466A>G	p.Tyr489Cys	Missense/Splicing	LOVD(reported 34 times)	r.1466_1527del62	Benigh	Tolerated	Disease causing
E17	c.1885G>A	p.Gly629Arg	Missense/Splicing	CM961026	r.1846_1886del41	Probably damaging	Tolerated	Disease causing
E20 (2)#	c.2329T>G	p.Trp777Gly	Missense	LOVD	NA	Probably damaging	Deleterious	Disease causing
E25	c.3211G>C	p.Ala1071Pro	Missense	This report	NA	Probably damaging	Deleterious	Disease causing
E34(2)	c.4402 A>G	p.Ser1468Gly	Missense/Splicing	LOVD(reported 3 times)	**r.4368_4402del35**	Benigh	Tolerated	Disease causing
E35	c.4574 T>C	p.Leu1525Pro	Missense	LOVD	NA	Probably damaging	Deleterious	Disease causing
E45	c.6755A>G	p.Lys2252Arg	Missense	LOVD (Pathogenic)	NA	Benigh	Tolerated	Disease causing
E48	c.7126G>A	p.Gly2376Arg	Missense/Splicing	This report	NA	Possibly damaging	Deleterious	Disease causing

Location	Nucleotide change	Amino acid change	Variant type	References	mRNA effect
In02	c.204+1G>A	p.Val34_Met68del	Splicing	CS031782	r.100_204del105
In03	c.288+1G>T	p.Arg69_Gly96del	Splicing	CS034311	**r.205_288del84**
In10	c.1185+2T>A	NA	Splicing	This report	NA
In13	c.1527+1G>A	p.Ser465_Cys509del	Splicing	CS993383	**r.1393_1527del135**
In15 (2)	c.1721+3A>G	p.Ser574fs	Splicing	LOVD (reported 6 times)	**r.1528_1721del114**
In17	c.2002-2A>G	NA	Splicing	LOVD	NA
In18	c.2251+3A>T	NA	Splicing	This report	NA
In23	c.3113+2T>C	p.Tyr998_Arg1038del	Splicing	CS086415	**r.2991_3113del123**
In27	c.3708+1delG	NA	Splicing	This report	NA
In36	c.4773-2A>C	p.Phe1592fs	Splicing	CS086425	r.4773_5065del293 **NMD**
In37	c.5206-2A>G	p.Arg1849fs	Splicing	CS002460	**r.5206_5546del341**
In42	c.6364+3A>T	p.Val2029fs	Splicing	Sabbagh et al., 2013	r.6085_6364del280
In53	c.7806+1G>A	NA	Splicing	LOVD	NA
Location	Nucleotide change	Amino acid change	Variant type	References	mRNA effect
E04	c.441C>A	p.Cys147*	Nonsense	This report	**r.441c>a**
E05	c.571A>T	p.Lys191*	Nonsense	Sabbagh et al., 2013	**r.571a>u**
E05 (2)#	c.574C>T	p.Arg192*	Nonsense	LOVD(reported 22 times)	r.574c>u
E06	c.616A>T	p.Lys206*	Nonsense	This report	NA
E08	c.808C>T	p.Gln270*	Nonsense	This report	NA
E09 (2)	c .910C>T	p.Arg304*	Nonsense	CM087437	r.910c>u, r.889_1062del174

E12	c.1318C>T	p.Arg440 *	Nonsense	LOVD(reported 18 times)	**r.1318c>u**
E14	c.1627C>T	p.Gln543 *	Nonsense	CM020463	r.1627c>u
E15 (2)#	c.1702G>T	p.Glu568 *	Nonsense	This report	r.1702g>u
E18 (2)	c.2041C>T	p.Arg681*	Nonsense	LOVD(reported 20 times)	r.2041c>u
E21	c.2620A>T	p.Lys874 *	Nonsense	This report	NA
E21	c.2848C>T	p.Gln950 *	Nonsense	This report	NA
E27	c.3562C>T	p.Gln1188 *	Nonsense	LOVD	NA
E28	c.3826C>T	p.Arg1276 *	Nonsense	LOVD(reported 22 times)	r.3826c>u
E35	c.4537C>T	p.Arg1513*	Nonsense	CM941093	r.4537c>u
E37	c.4786C>T	p.Gln1596*	Nonsense	This report	**r.4786c>u**
E39	c.5594T>G	p.Leu1865 *	Nonsense	This report	NA
E45	c.6709C>T	p.Arg2237 *	Nonsense	CM000815	r.6709c>u
E51	c.7486C>T	p.Arg2496 *	Nonsense	LOVD(reported 16 times)	r.7486c>u
Location	Nucleotide change	Amino acid change	Variant type	References	mRNA effect
E02 #	c.162_163insAT	p.Ser56fs	Frameshift	This report	NA
E03	c.233dupA	p.Asn78fs	Frameshift	LOVD	NA
E04	c.393_403delAGCTGAACTTC	p.Ala131fs	Frameshift	This report	NA
E05	c.496_497delGT	p.Val166fs	Frameshift	CD000948	**r.496_497delgu**
E08	c.779_795delCCAAACGTAAAGCAGCA	p.Thr260fs	Frameshift	This report	NA

E14	c.1541_1542delAG	p.Gln514fs	Frameshift	CD962097	r.1541_1542delag
E15	c.1676_1677insA	p.Asp559fs	Frameshift	This report	**r.1676_1677insa**
E18	c.2018_2019insTCGG	p.Ser674fs	Frameshift	This report	NA
E18	c.2230_2231delGT	p.Val744fs	Frameshift	This report	NA
E21	c.2828dupA	p.Phe944fs	Frameshift	LOVD	NA
E22	c.2855delT	p.Leu952fs	Frameshift	LOVD	NMD?
E27	c.3525_3526delAA	p.Arg1176fs	Frameshift	CD000971	NA
E29	c.3947_3948delAT	p.His1316fs	Frameshift	This report	NA
E30	c.4069_4072delTTCCinsCT	p.Phe1357fs	Frameshift	This report	NA
E32	c.4154delG	p.Gly1385fs	Frameshift	This report	**r.4154delg**
E36	c.4751delT	p.Tyr1586fs	Frameshift	This report	NA
E37 (2)	c.4914_4917delCTCT	p.Ser1660fs	Frameshift	LOVD(reported 3 times)	NA
E37	c.4781delC	p.Thr1594fs	Frameshift	This report	NA
E39	c.5717delT	p.Leu1906fs	Frameshift	This report	**r.5717delu**
E42	c.6336_6337delGT	p.Cys2113fs	Frameshift	This report	NA
E43	c.6527_6528delTT	p.Phe2176fs	Frameshift	LOVD	NA
E43	c.6470delT	p.Phe2157fs	Frameshift	CD000990	NMD?
E44	c.6597delT	p.Pro2200fs	Frameshift	This report	**r.6597delu**
E46	c.6791dupA	p.Tyr2264fs	Frameshift	LOVD(reported 10 times)	r.6757_6858del102 NMD r.6791dupa
E48	c.7120_7121dupTT	p.Leu2374fs	Frameshift	This report	r.7120_7121dupuu

Location	Nucleotide change	Amino acid change	Variant type	References	mRNA effect
E02 (2)#	c.177_179delTAC	p.60delThr	Inframe Deletion	This report	NA
E11	c.1250_1256delinsAAGA	p.Thr417fs	Delins	This report	NA
E39	c.5565_5567delTCT	p.Leu1856fs	Inframe Deletion	This report	NA
E40 (2)#	c.5943G>A	p.Gln1981Gln	Silent/Splicing	CS072261	r.5940_5943del4

The number in '( )' is the recurrences of mutations in this cohort. '#' means an inherited mutation.
NA: Not available. LOVD: data available on website(http://www.LOVD.nl/NF1) NMD: Nonsense-mediated mRNA decay
mRNA effect in bold：the RNA effect also has been demonstrated in our RNA test.
PolyPhen2(http://genetics.bwh.harvard.edu/pph2) , SIFT (http://sift.bii.a-star.edu.sg) and Mutation Taster(http://www.mutationtaster.org/) were applied to predict the potential effect of an amino acid substitution on the structure and function of the NF1 protein.

Table S2 .Prevalences of NF1 clinical features by age category

	Number and percentage of patients by age category (years)				
	0-3(n=18)	4-8(n=18)	9-19(n=21)	>19(n=40)	Total(n=97)
Fulfilled NF1 criteria	16.7%(3/18)	88.9(16/18)	95.2%(20/21)	97.5%(39/40)	80.4%(78/97)
**Major Clinical feature**					
Neurofibromas	5.6%(1/18)	33.3%(6/18)	71.4%(15/21)	92.5%(37/40)	59.8%(58/97)
Skin-fold freckling	0%(0/18)	50%(9/18)	68.8%(11/16)	70%(28/40)	49.5%(48/97)
Lisch nodules	25%(1/4)	69.2%(9/13)	66.7%(4/6)	100%(3/3)	65.4%(17/26)
Optic glioma	0%(0/5)	8.3%(1/12)	0%(0/3)	—	5%(1/20)
Osseous lesions	0%(0/18)	11.1%(2/18)	21.4%(3/14)	11.1%(4/36)	10.5%(9/86)
**Minor Clinical feature**					
Neuropsychological abnormalities	11.1%(1/9)	17.6%(3/17)	40%(2/5)	7.1%(1/14)	15.6%(7/45)
Malignancy	5.6%(1/18)	0%(0/18)	0%(0/17)	19.4%(7/36)	9%(8/89)
UBOs	40%(2/5)	58.3%(7/12)	66.7%(2/3)	—	55%(11/20)
Anemic nevus					7.4%(4/54)

Neurofibromas include cutaneous or subcutaneous neurofibromas, plexiform neurofibromas.
Osseous Lesions include macrocephaly, facial dysmorphism, scoliosis, tibial dysplasia.
The percentages were calculated by considering only those patients with corresponding clinical data.

Table S3.The molecular results and clinical details of 109 index patients

Patient No.	Gender /age	Family History	Nucleotide Change	Mutation Type	Phenotype							
					CALs (onset age)	Skin-fold freckling	NF (onset age)	Ocular abnormalities	Malignancy	Osseous lesions	Neuropsychological abnormalities	Special features
**Patients without neurofibromas and neoplasms**												
NF009	F/9y	—	c.1250_1256delinsAAGA	Frameshift	MC (birth)	Axilla Groin	—	?	—	?	?	?
NF010	M/5y	—	c.7120_7121dupTT	Frameshift	MC (1m)	—	—	LN	—	—	—	UBOs
NF012	F/4y	—	c.3211G>C	Missense	MC (birth)	—	—	?	—	—	—	?
NF013	M/10y	—	—	—	MC (birth)	—	—	?	—	—	—	—
NF014	M/9y	—	c.1541_1542delAG	Frameshift	MC (birth)	Axilla Groin	—	LN	—	—	BP	UBOs
NF017	F/3y	—	—	—	MC (birth)	—	—	—	—	—	—	UBOs

NF018	M/2y	—	c.4914_4917delCTCT	Frameshift	MC (birth)	—	—	?	—	—	—	Hemangioma
NF019	M/2y	—	c.4773-2A>C	Splicing	MC (birth)	—	—	?	—	—	—	?
NF024	F/2y	—	c.4154delG	Frameshift	MC (birth)	—	—	?	—	—	—	—
NF034	F/0y	—	—	—	MC (birth)	—	—	?	—	—	?	?
NF036	F/4y	—	—	—	MC (birth)	—	—	?	—	—	—	?
NF037	F/4y	Father (NF128)	—	—	MC (birth)	—	—	?	—	—	—	?
NF039	M/0y	—	c.1318C>T	Nonsense	MC (birth)	—	—	?	—	—	?	?
NF044	F/0y	—	c.7486C>T	Nonsense	MC (birth)	—	—	?	—	—	?	?
NF045	F/1y	—	c.2855delT	Frameshift	MC (1m)	—	—	?	—	—	—	?
NF047	M/10y	—	c.3708+1delG	Frameshift	MC (birth)	Axilla Groin	—	LN	—	—	—	AN
NF048	M/0y	—	—	—	MC (birth)	—	—	?	—	—	?	?

NF049	F/2y	—	—	—	MC (birth)	—	—	—	—	—	—	—
NF050	M/0y	—	c.2018_2019insTCGG	Frameshift	MC (birth)	—	—	?	—	—	?	?
NF052	F/1y	—	c.496_497delGT	Frameshift	MC (birth)	—	—	?	—	—	—	?
NF053	F/3y	—	c.571A>T	Nonsense	MC (birth)	—	—	LN	—	—	—	UBOs
NF070	M/38y	—	c.2230_2231delGT	Frameshift	MC (?)	—	—	?	—	—	?	?
NF089	F/19y	+	c.746T>C	Missense	MC (birth)	—	—	?	—	—	—	?
NF093	F/2y	—	c.233dupA	Frameshift	MC (birth)	—	—	—	—	—	—	JX
NF094	F/5y	—	type-1 deletion	Microdeletion	MC (birth)	Axilla Groin	—	Strabismus	—	—	MR BP SP	UBOs
NF096	M/8y	—	c.5206-2A>G	Splicing	MC (birth)	—	—	LN	—	—	—	UBOs
NF097	M/0y	—	c.1527+1G>A	Splicing	MC (birth)	—	—	?	—	—	?	?
NF099	M/10y	?	c.204+1G>A	Splicing	MC (birth)	Axilla Groin	—	?	—	—	?	?
NF106	M/0y	—	c.1721+3A>G	Splicing	MC (10d)	—	—	?	—	—	?	AN

NF108	M/4y	—	c.1676_1677insA	Frameshift	MC (10d)	—	—	LN	—	—	—	UBOs
NF109	F/0y	—	c.5717delT	Frameshift	MC (birth)	—	—	?	—	—	?	?
NF113	M/5y	—	—	—	MC (birth)	Axilla	—	—	—	—	—	—
NF114	M/3y	—	c.4574T>C	Missense	MC (birth)	—	—	—	—	—	—	AN
NF115	F/4y	—	c.6755A>G	Missense	MC (birth)	Axilla	—	—	—	—	—	—
NF116	M/5y	—	c.3947_3948delAT	Frameshift	MC (birth)	Axilla Groin	—	LN	—	—	—	?
NF117	M/9y	—	—	—	MC (birth)	Groin	—	LN	—	—	—	—
NF118	F/0y	—	c.1721+3A>G	Splicing	MC (birth)	—	—	?	—	—	?	—
NF121	M/0y	—	c.3113+2T>C	Splicing	MC (birth)	—	—	?	—	—	?	?
NF122	M/7y	—	c.2251+3A>T	Splicing	MC (birth)	Axilla	—	LN	—	—	—	—
**Patients with neurofibromas or neoplasms**												
NF002	M/12y	—	c.4781delC	Frameshift	MC (birth)	Axilla Groin	CNF,SCNF,PNF	LN	—	MA	MR, SP,BP	RVH

							(2y)						
NF020	F/8y	—	c.1185+2T >A	Splicing	MC (birth)	—	PNF (?)	?	—	FD,SCS	—	?	
NF021	F/10y	—	type-1 deletion	Microdeletion	MC (birth)	Axilla Groin	SCNF,PNF (1y)	Amblyopia	—	FD	—	?	
NF023	M/11y	—	type-1 deletion	Microdeletion	MC (birth)	Axilla Groin	SCNF,PNF (birth)	LN	—	FD	SP	UBOs	
NF025	F/45y	—	—	—	MC (birth)	Axilla Groin	CNF,SCNF,PNF (20y)	?	Teratoma (?)	SCS	—	?	
NF026	F/39y	—	c.737C>A	Missense	MC (7y)	Axilla Groin	—	?	MNPST (?)	—	—	?	
NF027	M/44y	father	c.4402A>G	Missense	MC (birth)	Axilla Groin	CNF,SCNF (10y)	LN	—	—	—	?	
NF028	F/14y	?	c .910C>T	Nonsense	MC (?)	?	+ (?y)	?	?	?	?	?	
NF030	F/15y	—	c .910C>T	Nonsense	MC (?)	?	+ (?y)	?	?	?	?	?	
NF031	F/17y	?	c.393_403 delAGCT GAACTT	Frameshift	MC (?y)	?	+ (?y)	?	?	?	?	?	

			C								
NF032	M/22y	+	c.2329T>G	Missense	MC (2y)	Axilla Groin	CNF,SCNF (20y)	?	MPNST (?)	—	—	?
NF033	M/56y	+	c.162_163 insAT	Frameshift	MC (birth)	Axilla Groin	CNF,SCNF (?y)	?	—	SCS	MR	?
NF035	F/15y	?	c.4402 A>G	Missense	MC (?)	?	CNF,SCNF (?y)	?	—	?	?	?
NF040	F/1y	—	Atypical deletion	Microdeletion	MC (birth)	—	PNF (birth)	?	—	—	?	?
NF042	F/4y	—	c.6791dupA	Frameshift	MC (birth)	Axilla	SCNF (2y)	LN	—	—	—	UBOs
NF054	M/4y	Mother (NF130)	c.177_179 delTAC	Inframe deletion	MC (birth)	—	—	OG	—	—	—	UBOs
NF055	M/26y	—	c.2620A>T	Nonsense	MC (6y)	—	—	?	—	—	?	?
NF056	F/60y	—	Atypical deletion	Microdeletion	MC (birth)	Groin	+ (26y)	?	—	—	?	?
NF058	F/44y	—	c.2041C>T	Nonsense	MC (?)	—	+ (18y)	?	—	—	?	?

NF059	M/20y	—	c.6527_6528delTT	Frameshift	MC (2y)	Axilla Groin	+ (9y)	?	—	—	?	?
NF060	M/58y	Daughter(NF125)	c.574C>T	Nonsense	MC (birth)	Axilla	+ (30y)	?	—	—	?	?
NF061	M/25y	—	c.2002-2A>G	Splicing	MC (birth)	Axilla Groin	+ (6y)	?	—	—	?	?
NF062	F/17y	Mother (NF126)	type-1 deletion	Microdeletion	MC (birth)	—	+ (13y)	?	—	MA	?	?
NF063	M/21y	—	c.3562C>T	Nonsense	MC (birth)	Axilla Groin	+ (15y)	?	—	—	?	?
NF064	F/41y	Son(NF129)	c.5943G>A	Silent	MC (?)	Axilla	+ (26y)	?	—	—	?	?
NF065	M/19y	—	c.4537C>T	Nonsense	MC (birth)	Axilla	+ (16y)	?	—	—	?	?
NF066	M/33y	—	c.5565_5567delTCT	Frameshift	MC (birth)	Axilla	+ (20y)	?	—	—	?	?
NF067	F/16y	—	c.2848C>T	Nonsense	MC (birth)	Axilla Groin	+ (7y)	?	—	—	?	?
NF068	M/55y	—	c.4069_4072delTTCCinsCT	Frameshift	MC (?)	—	+ (11y)	?	—	—	?	?
NF069	F/38y	—	c.1627C>T	Nonsense	MC (?)	—	+ (16y)	?	—	—	?	?

NF071	M/27y	—	c.4751delT	Frameshift	MC (birth)	—	+ (15y)	?	—	—	?	?
NF072	M/20y	—	type-1 deletion	Microdeletion	MC (1y)	—	+ (1y)	?	—	FD	?	Acromegalia
NF073	F/25y	—	Atypical deletion	Microdeletion	MC (birth)	Groin	+ (8y)	?	—	—	?	?
NF074	F/13y	—	c.6336_6337delGT	Frameshift	MC (birth)	—	+ (9y)	?	—	—	?	?
NF075	M/6y	—	c.2828dupA	Frameshift	MC (birth)	Groin	+ (2y)	?	—	—	?	?
NF076	F/36y	—	Atypical deletion	Microdeletion	MC (birth)	—	+ (6y)	?	—	—	?	?
NF077	F/42y	—	c.808C>T	Nonsense	MC (?)	—	+ (25y)	?	—	—	?	?
NF078	M/45y	—	Type 2 deletion	Microdeletion	MC (1y)	—	+ (14y)	?	—	SCS	?	?
NF079	M/12y	—	c.779_795delCCAAACGTAAAGCAGCA	Frameshift	MC (birth)	—	+ (2y)	?	—	—	?	?
NF080	M/13y	—	c.1885G>A	Missense	MC (birth)	—	+ (birth)	?	—	—	?	?

NF081	F/22y	—	c.4914_4917delCTCT	Frameshift	MC (?)	—	+ (17y)	?	—	—	?	?
NF082	M/22y	—	c.3826C>T	Nonsense	MC (birth)	Axilla Groin	+ (18y)	?	—	—	?	?
NF084	F/18y	—	—	—	MC (?)	—	+ (?)	?	Oral neoplasms (?y)	FD	?	Acromegalia
NF085	F/47y	—	c.2041C>T	Nonsense	MC (birth)	Axilla Groin	+ (3y)	?	—	—	?	?
NF086	F/23y	—	c.7126G>A	Missense	MC (5y)	Axilla	+ (7y)	?	—	—	?	?
NF087	M/22y	—	type-1 deletion	Microdeletion	MC (1y)	Axilla	+ (12y)	?	—	—	?	?
NF088	F/26y	—	c.6709C>T	Nonsense	MC (8y)	—	+ (22y)	?	—	TD	?	?
NF095	M/43y	Daughter (NF127)	E28 deletion	Single exon deletion	MC (birth)	Axilla Groin	CNF, SCNF, PNF (20y)	LN	—	—	—	?
NF098	M/5y	—	c.6597delT	Frameshift	MC (birth)	Axilla	PNF (birth)	LN	—	FD	SP,BP MR	—
NF100	M/5y	Mother (NF123)	c.1702G>T	Nonsense	MC (birth)	Groin	CNF (4y)	?	—	—	—	?

NF101	M/29y	—	c.5594T>G	Nonsense	MC (birth)	Axilla Groin	PNF (?y)	?	—	—	—	?
NF102	F/49y	—	c.3525_3526delAA	Frameshift	MC (birth)	Axilla Groin	CNF, SCNF, PNF (1y)	?	GIST (48y)	—	—	?
NF103	F/26y	Farther (NF124)	c.7806+1G>A	Splicing	MC (2y)	Axilla Groin	CNF, SCNF (12y)	?	MNPST (24y)	—	—	?
NF104	F/25y	—	c.1466A>G	Missense	MC (2y)	Axilla Groin	CNF, SCNF (7y)	?	MRMS (25y)	—	—	?
NF105	M/50y	—	c.6364+3A>T	Splicing	MC (5y)	Axilla Groin	CNF, SCNF (5y)	?	GIST (50y)	—	—	?
NF107	F/3y	—	c.6470delT	Frameshift	MC (birth)	—	PNF (birth)	—	Neuroblastoma (?)	—	SP, MR,BP	UBOs
NF110	M/4y	—	c.1207C>T	Missense	MC (birth)	Groin	PNF (birth)	—	—	—	—	AN
NF111	F/8y	—	c.616A>T	Nonsense	MC (birth)	—	PNF (birth)	—	—	—	—	UBOs
NF112	M/5y	—	c.288+1G>T	Splicing	MC (birth)	—	SCNF, PNF (5y)	LN	—	—	SP	—

NF119	F/4y	—	c.441C>A	Nonsense	MC (birth)	—	SCNF (1y)	LN	—	—	—	?
NF120	M/13y	Mother (NF131)	c.4786C>T	Nonsense	MC (birth)	Axilla Groin	PNF (13y)	—	—	—	—	—
**Index family members**												
NF123	F/26y	Son (NF100)	c.1702G>T	Nonsense	MC (birth)	Axilla Groin	+ (?y)	?	?	?	—	?
NF124	M/49y	Daughter (NF103)	c.7806+1G>A	Splicing	MC (?)	Axilla Groin	+ (?y)	?	?	?	?	?
NF125	F/37y	Father (NF060)	c.574C>T	Nonsense	MC (birth)	Axilla Groin	+ (18y)	?	?	?	—	?
NF126	F/40y	Daughter (NF062)	type-1 deletion	Microdeletion	MC (?)	?	+ (?)	?	?	?	?	?
NF127	F/12y	Father (NF095)	E28 deletion	Single exon deletion	MC (birth)	Axilla Groin	—	—	—	—	—	?
NF128	M/27y	Daughter	—	—	MC (birth)	Axilla Groin	CNF, SCNF	?	—	—	—	?

		(NF037)					(?)					
NF129	M/19y	Mother (NF064)	c.5943G>A	Silent	MC (birth)	Axilla Groin	+ (19y)	?	—	?	?	?
NF130	F/25y	Son (NF054)	c.177_179 delTAC	Inframe deletion	MC (birth)	Axilla Groin	CNF, SCNF (19y)	LN	—	—	—	?
NF131	F/36y	Son (NF120)	c.4786C>T	Nonsense	MC (birth)	Axilla Groin	CNF, SCNF (30y)	?	—	—	—	?

"+" means positive result; "—" means negative result; "?" means unclear result.

CALs: Cafè-au-Lait Spots, MC: Multiple CALs. AF: Axillary Freckling. LN: Lisch Nodules.

CNf: Cutaneous Neurofibromas, SCNf: Subcutaneous Neurofibromas, PNf: Plexiform Neurofibromas.

MA: Macrocephaly, FD: Facial dysmorphism, SCS: Scoliosis, TD: Tibial dysplasia.

MR: Mental Retardation, SP: Speaking problems, BP: Behavior problems.

MPNST: Malignant peripheral nerve sheath tumor, CG: Cerebral glioma, GIST: Gastrointestinal stromal tumor, MRMS: Mediastinal rhabdomyosarcoma

UBOs: Unidentified Bright Objects, AN: Anemic nevus, JX: Juvenile xanthogranuloma, RVH: Renal vascular hypertension.

Age <1 year was denoted by '0y'.

Table S4. List of the identified microdeletion in MLPA

**Probe**	**Patient No.**											
	NF021	NF023	NF040	NF056	NF062	NF072	NF073	NF076	NF078	NF087	NF094	NF126
TRAF4 - 2	—	—	—	—	—	—	—	—	—	—	—	—
TRAF4 - 4	—	—	—	—	—	—	—	—	—	—	—	—
SSH2 - 14	—	—	—	—	—	—	—	—	—	—	—	—
SSH2 - 4	—	—	—	—	—	—	—	—	—	—	—	—
BLMH - 9	—	—	—	—	—	—	—	—	—	—	—	—
BLMH - 2	—	—	—	—	—	—	—	—	—	—	—	—
CPD - 11	—	—	—	—	—	—	—	—	—	—	—	—
CPD - 20	—	—	—	—	—	—	—	—	—	—	—	—
SUZ12P - up	+	+	—	—	+	+	—	—	+	+	+	+
SUZ12P - 01	+	+	—	—	+	+	—	—	+	+	+	+
SUZ12P - 06	+	+	—	+	+	+	—	+	+	+	+	+
CRLF3	+	+	—	+	+	+	—	+	+	+	+	+
ATAD5	+	+	—	+	+	+	—	+	+	+	+	+
ADAP2	+	+	—	+	+	+	—	+	+	+	+	+
RNF135	+	+	—	+	+	+	—	+	+	+	+	+

NF1 - 1	+	+	+	+	+	+	+	+	+	+	+	+
NF1 - 17	+	+	+	+	+	+	+	+	+	+	+	+
NF1 - 30	+	+	+	+	+	+	+	+	+	+	+	+
NF1 - 49	+	+	+	+	+	+	+	+	+	+	+	+
NF1 - 57	+	+	+	+	+	+	+	+	+	+	+	+
UTP6	+	+	+	—	+	+	+	+	+	+	+	+
SUZ12	+	+	+	—	+	+	+	+	+	+	+	+
LRRC37B	+	+	+	—	+	+	+	+	—	+	+	+
ZNF207	—	—	+	—	—	—	—	—	—	—	—	—
PSMD11 - 2	—	—	—	—	—	—	—	—	—	—	—	—
PSMD11 - 6	—	—	—	—	—	—	—	—	—	—	—	—
MYO1D - 7	—	—	—	—	—	—	—	—	—	—	—	—
MYO1D - 2	—	—	—	—	—	—	—	—	—	—	—	—
PRS1	—	—			—	—				—	—	—
PRS2	+	+			+	+				+	+	+
Distance of Deletions (mb)	1.4	1.4	1.27-1.46	0.60-1.14	1.4	1.4	0.93-1.28	1.26-1.63	1.2	1.4	1.4	1.4
Type of Deletions	type 1 deletions	type 1 deletions	atypical deletions	atypical deletions	type 1 deletions	type 1 deletions	atypical deletions	atypical deletions	type 2 deletions	type 1 deletions	type 1 deletions	type 1 deletions

Peak area value less than 0.7 was suggestive of deletions/positive result.

Table S5. List of the identified non-pathogenic polymorphisms

Patient No.	Location	Genomic variant(status)	Type	NF1 causing mutation	Reference
NF034	E02	c.168C>T(hetero)	Synonymous variant	Not found	rs17881168
NF036<br>NF059<br>NF102	E04	c.369C>G(hetero)	Synonymous variant	Not found<br>c.6527_6528delTT<br>c.3525_3526delAA	rs146691765
common	E07	c.702G>A(hetero,homo)	Synonymous variant	_	rs1801052
NF017	E10	c.1089A>C(hetero)	Synonymous variant	Not found	Novel
NF012<br>NF030<br>NF033<br>NF037	E17	c.1933A>G(hetero)	Missense variant	c.3211G>C<br>c .910C>T<br>c.162_163insAT<br>Not found	rs146051850<br>tolerated (Sift)<br>benigh(Polyphen2<br>)
NF048	E14	c.1581A>C(homo)	Synonymous variant	Not found	Novel
common	E18	c.2034G>A(hetero,homo)	Synonymous variant	_	rs2285892
NF105	E56	c.8088G>A(hetero)father	Synonymous variant	c.6364+3A>T	rs2285895
NF044	E50	c.7305A>G(hetero)	Synonymous variant	c.7486C>T	rs201287021
common	In12	c.1393-32T>C(hetero,homo)	Intron variant	-	rs2905876
common	In14	c.1641+39T>C(hetero,homo)	Intron variant	_	rs2905880

common	In26	c.3496+33C>A(hetero,homo)	Intron variant	_	rs2066736
common	In37	c.5205+23T>C(hetero,homo)	Intron variant	_	rs9894648
common	In38	c.5546+19A>T(hetero,homo)	Intron variant	_	rs2285894
common	In43	c.6579+45T>A(hetero,homo)	Intron variant	_	rs17883614
NF024<br>NF049	In46	c.6858+40A>G(hetero,homo)	Intron variant	_<br>Not found	rs188244160
common	In48	c.7126+37C>G(hetero,homo)	Intron variant	_	rs7405740

Table S6. List of the primers of *NF1*

Primer name	Primer Sequence	Product Size (bp)
**NF1-E01up_F**	TCGGACTGTGATGGCTGTGGG	1271
**NF1-E01up_R**	TTGCTCTGTTGTCGGTGGCTC	
**NF1-E02up_F**	TCTCATGGGATTTGTCTGCTT	1187
**NF1-E02up_R**	ATCTACTTAGGGCTGGGACAA	
**NF1-E03up_F**	TAATGGCAGACTCTAATAAATG	775
**NF1-E03up_R**	ATAAAACAAGTGTTCTCAAGG	
**NF1-E04up_F**	AATGGGAGGACTGGTAAGGAT	937

**NF1-E04up_R**	GTGATAAAGGGTAGAGTTGTG	
**NF1-E05up_F**	GAGATCCTCCTCCCTTAGCCT	633
**NF1-E05up_R**	ATGAAATAATAGCACCCTTGC	
**NF1-E06+07_F**	TTTTGGGTTTATGTTAGGTGT	598
**NF1-E06+07_R**	AACTTGGAAAACGATGATAGG	
NF1-E08_F	ATTACATTGCTTGTCTACTTAC	437
NF1-E08_R	TTCCCTGAAATACTTTGCTTA	
**NF1-E09up_F**	GGCAACAGAGGAAGACACCAT	807
**NF1-E09up_R**	AGAAGCACAAAGCACCCATAC	
**NF1-E10+11_F**	GTATGGGTGCTTTGTGCTTCT	1774
**NF1-E10+11_R**	TCCCTCAGGCATGTTTATTTG	
**NF1-E12up_F**	AAATTCACTGGCTAATCATCG	772
**NF1-E12up_R**	AGTCAGCTTCCCTTACTCATC	
**NF1-E13up_F**	AAAAGAGGTGGAGTGCCGTCG	626
**NF1-E13up_R**	ATGGCTTGAGGTCAGGAGTTCG	
**NF1-E14up_F**	CTCCCAAAGTCCTGGGCTTAC	1144
**NF1-E14up_R**	CCAAACGTCCACCAACTGATA	
**NF1-E15up_F**	ATTCTGTCTGTATTATTCCCT	463
**NF1-E15up_R**	ATTGCTGTGATACTTTTCTTA	
**NF1-E16up_F**	ATAACCATTCAGTTCTGCCTTTG	778
**NF1-E16up_R**	TGATTTGCCAGTCATTGTCCT	
**NF1-E17up_F**	ATAGACTAATGGTGAAGTGGGTT	924
**NF1-E17up_R**	CTTCTGTGGTATGAATGGGTT	
NF1-E18_newF	CACAGTTTATTGCATTGTTAGAT	380
NF1-E18_newR	GCCATGTGCTTTGAGGCAGAC	

NF1-E19+20_newF	TTGCTCTGCTCTTCCTACTCCT	592
NF1-E19+20_newR	ATATGTTTACTTTACTGAGCGACTCT	
NF1-E21_newF	TGGATAAAGCATAATTTGTCAAGT	549
NF1-E21_newR	TAGAGAAAGGTGAAAAATAAGAG	
NF1-E22_newF	CTCTGTGTGTTTAGATCAGTCA	319
NF1-E22_newR	TTTATCAATTACTACCAGTAGCAG	
NF1-E23_newF	GGTGTGACAGTAAGGTAGCCAGA	386
NF1-E23_newR	TCCTTTCTACCAATAACCGCAT	
NF1-E24_newF	TCATGTCACTTAGGTTATCTGG	270
NF1-E24_newR	TGTAATTAAGTAGTTATAACTCTC	
NF1-E25_newF	AAAGAACTTGAAAGATTCATGGTCTC	267
NF1-E25_newR	TCCTGTCATGGGTATTATTTCTTT	
NF1-E26+27_newF	GTAAAATCATGTCCAACATAGCACA	831
NF1-E26+27_newR	CCTAACTAAACATCTTTCTTCTG	
**NF1-E28+29up_F**	ATTCAGTATTCGCTGAGTTCC	1247
**NF1-E28+29up_R**	TACCTATTAGCCTCTTTCCCT	
**NF1-E30up_F**	GAGATTCAGTCTTGCCCTTTC	853
**NF1-E30up_R**	TTTCAGCCACATCCATCATAC	
**NF1-E31up_F**	TGTGATTCATAGCCAGAAATAG	653
**NF1-E31up_R**	AAGACAGTCAGCGTTTCCTAA	
NF1-E32+33_newF	GGTCTGCTTTCATTACTCATC	1400
NF1-E32+33up_R	TCTTCCTTGTCCACATATTGT	
NF1-E34+35up_F	CCCTCCATATTTGTAATCTTAG	1885
NF1-E34+35up_R	ATAATGTTGTCTTCACTCCCT	
**NF1-E36_newF**	CCAAATTACCCTTTAGAATGCCT	500

**NF1-E36_R**	GCTACTAGATACCGACCATCA	
**NF1-E36_F**	AGACAACATAAAGCCTCATAA	300
**NF1-E36_newR**	CTTCAATTCCTGTTAAGTCAACTGG	
**NF1-E37up_F**	GCTCCAGATGGGTCATTCCTA	1254
**NF1-E37up_R**	ACGTCTCATATCCTCCACAGG	
**NF1-E38up_F**	TCTTCCTTAAATGGCATAGTGT	883
**NF1-E38up_R**	GTGATGTAGGTGGAAAGTATG	
**NF1-E39up_F**	TTAGGACACCCAGAAAGAAGT	994
**NF1-E39up_R**	CTTTGTAAGTGGTCCAGAGGT	
NF1-E40_F	ATAATTGTTGATGTGATTTTCATTG	424
NF1-E40_R	AATTTTGAACCAGATGAAGAG	
**NF1-E41+43up_F**	TCTGAAGGAGTCAAATGAATA	1900
**NF1-E41+43up_R**	GCTAATACACCCAAAGACAAC	
**NF1-E44+46up_F**	CATTGCCTTCCGTTCCAGTTA	1843
**NF1-E44+46up_R**	CACCCAAACTCTTATGACGAT	
**NF1-E47up_F**	GACTTAGCTCCTGGTGGTAGT	926
**NF1-E47up_R**	TGTATAGTGTTTAGCATACGG	
**NF1-E48up_F**	TTTCATCTTCCACCATCTTCT	672
**NF1-E48up_R**	TCCTTCCATCTATGCCTCCTA	
NF1-E49_F	ATTCACATATGCATGTTTTACCTTC	556
NF1-E49_R	GATAACAGAGAGCCACTGTAGTGTC	
NF1-E50_F	GTGCACATTTAACAGGTACTAT	373
NF1-E50_R	CTTCCTAGGCCATCTCTAGAT	
NF1-E51_F	CTTGGAAGGAGCAAACGATGGTTG	356
NF1-E51_R	CAAAAACTTTGCTACACTGACATGG	

**NF1-E52+54up_F**	AAACTGCTCCAGGGATGTATT	1870
**NF1-E52+54up_R**	AAGGTTGGCTCTGTCTGCTAC	
**NF1-E55+56up_F**	CACAATTTCTTCCCTGGTGAC	1258
**NF1-E55+56up_R**	AGCCTGGAGACAGAGTGAGAC	
**NF1-E55+56_newF**	TCATCTCCCTTTAATTTTGGCA	703
**NF1-E55+56_newR**	AACAGAAACCTTCATTTTGCCA	
**NF1-E57up_F**	CCAGTAACCCTCTGAGCCCTTTA	875
**NF1-E57up_R**	TTCCTCTTCCTGCCCAAACTC	
**NF1-E58up_F**	TATAGACAGTGGTCCCGAAAG	595
**NF1-E58up_R**	GGAAGTGCAGCATTACAACAT	

Primers in bold were used for nested PCR.

Figure S1. Relative mutation frequency over the *NF1* gene.

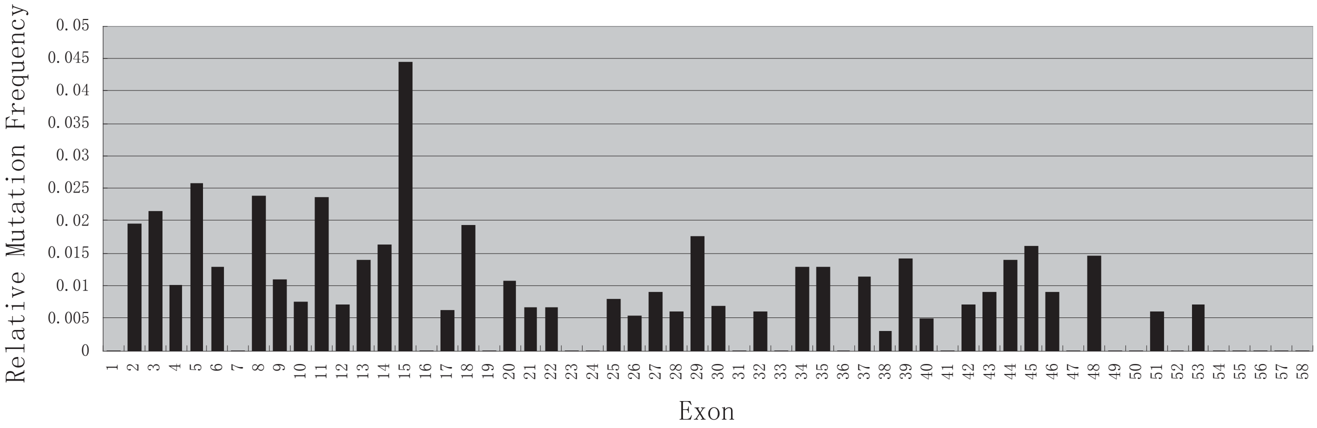

Splice site mutations were allocated to the nearest exon, ten base pairs (bp) were added to each exon, to allow for splice-site mutations. For each exon, the number of pathogenic mutations was divided by the number of bp.

REFERENCES

1. Kehrer-Sawatzki, H. et al. High Frequency of Mosaicism among Patients with Neurofibromatosis Type 1 (NF1) with Microdeletions Caused by Somatic Recombination of the JJAZ1 Gene. *Am J Hum Genet* **75**, 410-423 (2004).
2. Wimmer, K. et al. Spectrum of single-and multiexon NF1 copy number changes in a cohort of 1,100 unselected NF1 patients. *Genes, Chromosomes and Canc* **45**, 265-276 (2006).
3. Mautner, V.F. et al. Clinical characterisation of 29 neurofibromatosis type-1 patients with molecularly ascertained 1.4 Mb type-1 NF1 deletions. *J Med Genet* **47**, 623-30 (2010).
4. De Luca, A. et al. Deletions of NF1 gene and exons detected by multiplex ligation-dependent probe amplification. *J Med Genet* **44**, 800-8 (2007).
5. Valero, M.C. et al. A highly sensitive genetic protocol to detect NF1 mutations. *J Mol Diagn* **13**, 113-22 (2011).
